# Construction of a Molecularly Imprinted Sensor Modified with Tea Branch Biochar and Its Rapid Detection of Norfloxacin Residues in Animal-Derived Foods

**DOI:** 10.3390/foods12030544

**Published:** 2023-01-26

**Authors:** Shujuan Chen, Yiting Zhu, Jing Han, Tianyi Zhang, Runwen Chou, Aiping Liu, Shuliang Liu, Yong Yang, Kaidi Hu, Likou Zou

**Affiliations:** 1 College of Food Science, Sichuan Agricultural University, Ya’an 625014, China; 2 College of Resources, Sichuan Agricultural University, Chengdu 611130, China

**Keywords:** norfloxacin, biochar, molecular-imprinted polymer, double functional monomer, electrochemical trace detection

## Abstract

Norfloxacin (NOR) is a common antibiotic used in humans and animals, and its high levels can cause intolerance or poisoning. Therefore, NOR levels in animal-derived foods must be monitored due to potential side effects and illegal use phenomena. This research centered on the development of an environmentally friendly electrochemical sensor for NOR detection. Potassium carbonate activated tea branch biochar (K-TBC) as an efficient use of waste was coated on the surface of glassy carbon electrode (GCE), and a molecular-imprinted polymer (MIP) layer was subsequently electropolymerized onto the modified electrode. NOR was used as template molecule and o-phenylenediamine (o-PD) and o-aminophenol (o-AP) were used as bifunctional monomers. The electrochemical sensor was built and its electrochemical behavior on NOR was investigated. The sensor demonstrated an excellent linear current response to NOR concentrations in the ranges of 0.1–0.5 nM and 0.5–100 nM under ideal experimental circumstances, with a detection limit of 0.028 nM (S/N = 3). With recoveries ranging from 85.90% to 101.71%, the designed sensor was effectively used to detect NOR in actual samples of milk, honey, and pork. Besides, the fabricated sensor had low price, short detection time, good selectivity and stability, which can provide a theoretical and practical basis for the actual monitoring of NOR residues.

## 1. Introduction

Norfloxacin (NOR), a third-generation quinolone antibiotic, is a low-cost, highly effective broad-spectrum antimicrobial agent, which has been widely used in livestock and poultry farming and veterinary clinics [1,2]. However, NOR remains in meat, milk, honey, and other animal-derived foods, which may lead to pathogenic bacteria developing drug resistance, as well as joint pain, aortic aneurysms, and other diseases, posing a serious threat to human health [3,4,5]. In order to better guarantee the quality and safety of food of animal origin. China’s Ministry of Agriculture and Rural Affairs has issued a notice prohibiting the use of NOR in food animals [6]. However, some farmers or businesses continue to use banned antibiotics for profit. The results of veterinary drug residue detection of animal-derived foods in China (2020) showed that the residual amount of NOR was 163 μg/kg in honey samples, which seriously exceeded the standard [7]. As a result, NOR must be detected in animal-derived foods for risk assessment using appropriate, precise, and handy analytical methods.

At present, the conventional methods for NOR detection mainly include high performance liquid chromatography (HPLC) [8], high performance liquid chromatography–mass spectrometry (HPLC/MS) [9], microbial detection methods [10], and enzyme-linked immunosorbent assay (ELISA) [11]. These chromatographic methods have high detection accuracy and sensitivity, but they usually take a long time and require expensive and sophisticated analytical instruments. Meanwhile, the antigens and antibodies used in ELISA are expensive and easily cross-react, and microbial detection methods suffers from a high level of detection error, which significantly limits their widespread application. In contrast, the electrochemical sensor was considered to be a sensitive electrochemical analytical method with simple equipment, short analysis time and good repeatability, which can easily realize online detection of target substances, and it has broad development prospect [12,13].

In the last few years, porous materials based on electrochemical sensors have been brought into focus. Amongst multifarious porous materials, biochar (BC) is a potential material for sensor preparation due to its unique physical chemical and green environmental protection. BC is a highly aromatic porous material obtained by carbonization and activation at high temperature using carbon sources such as animals and plants, typically has a large specific surface area, high porosity (mostly mesoporous or microporous), good electrical conductivity, and abundant oxygen-containing functional groups (-COOH, -OH) [14,15,16], indicating that BC has excellent potential as electrode modification materials to construct electrochemical sensors. As a kind of biomass raw material, the fixed carbon content of tea waste is generally more than 10% [17], which is similar to the commercialized coconut shell and walnut shell [18,19], indicating that tea waste has the application potential of biochar preparation. Furthermore, China, the world’s largest tea producer, can generate approximately 1 million tons of pruned tea branches (TB) annually, which is not effectively used [20]. Consequently, it is worthwhile to research the conversion of wasted tea branches into high-value biological products. However, there are few reports on the application of tea tree branch biochar (TBC) in the direction of electrochemical sensing, it is commonly used to remove tetracycline from wastewater [20,21]. However, small selective adsorption capacity may occur when TBC is used alone, molecularly imprinted polymers (MIPs) were introduced to further improve the selectivity of the electrochemical sensor for NOR.

The benefits of molecular imprinting and electrochemical techniques are combined in molecularly imprinted electrochemical sensors (MIECS) with high selectivity and rapid detection capabilities. By means of bulk polymerization, suspension or precipitation polymerization (PPm), molecular self-assembly electropolymerization (EPm), and other processes, MIPs have been deposited on MIECSs [22,23]. EPm has the advantage of allowing the thickness and density of the polymer layer to be controlled by selecting appropriate functional monomers and adjusting electrochemical parameters, which eliminates the drawbacks of conventional MIPs such as incomplete template removal and low binding capacity [24]. The choice of functional monomer is critical for the EPm construction of an MIP-based sensor. Recently, aniline-based copolymers (such as o-phenylenediamine) have been synthesized and applied to novel MIPs [24]. Since this particular copolymer has novel properties brought about by other monomers in addition to the properties of Poly-aniline (PAn). It is crucial to remember that adding aminophenol to PAn-based copolymers expand the copolymer’s pH range since the hydroxyl functional groups on the benzene ring can undergo reversible oxidation and reduction. For instance, a poly(o-phenylenediamine-co-o-aminophenol) (PoPD-oAP)/GCE sensor was used to detect ascorbic acid with an LOD of 36.4 μmol/L [25] and an MIP (PoPD-oAP)/PEDOT:PSS@Fc/SPCE electrochemical sensor with an LOD of 8.3 × 10^−8^ ng/mL in the presence of prostate specific antigen (PSA) [26]. Compared with the MIP of a single functional monomer, the molecularly imprinted copolymer exhibits stronger sensitivity and selectivity to the target molecule, which may be because multiple functional groups can be recognized or interact with the template molecule in various ways. Therefore, MIP based on copolymer synthesis is beneficial to the development of MIECSs.

**Figure 1:**
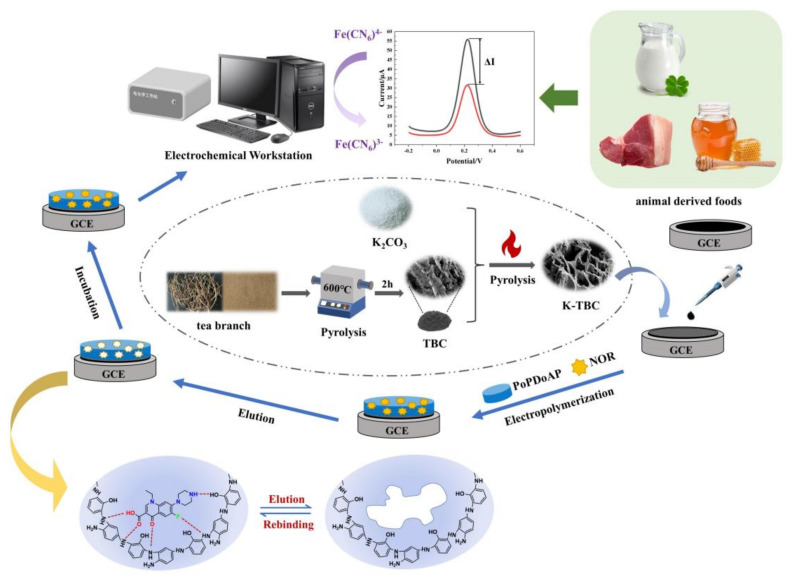
Schematic illustration of the sensor fabrication process and NOR detection.

Herein, a simple and efficient MIP electrochemical sensor for the detection of NOR was developed by stepwise modification of K-TBC and MIP films using binary functional monomers on glassy carbon electrodes, Figure 1 depicts the synthesis mechanism. Considering the superior porosity and effective catalytic activity, TBCs were prepared by facile pyrolysis and chemical activation methods to increase the sensor’s sensitivity to detection. By using the cyclic voltammetry (CV) approach, MIP films were electropolymerized onto TBC/GCE surfaces using o-PD and o-AP as binary functional monomers and NOR as a template molecule. Functional monomers and template molecules are connected by hydrogen bonding interactions because both o-PD and o-AP have amino groups. Therefore, the prepared MIP films have high template adsorption selectivity. The molar ratio of template to functional monomer, the proportion of bifunctional monomers, pH of polymerization solution, elution time, and incubation time were all optimized as experimental parameters affecting MIP electrochemical sensor performance. Furthermore, the sensor’s applicability in animal-derived food samples such as milk, honey, and pork were investigated. The proposed technology is the first use of an MIP electrochemical sensor by bifunctional monomers for NOR detection that we are aware of.

## 2. Materials and Methods

### 2.1. Apparatus and Reagents

Apparatus: The ZEISS Sigma 300 apparatus was used to perform scanning electron microscopy (SEM) at a voltage of 10.0 kV. The phase and elemental analyses were performed on an Ultima IV X-ray diffractometer (XRD, Rigaku, Saitama, Japan) and X-ray photoelectron spectroscope (XPS, K-Alpha, Thermo Scientific, Waltham, MA, USA). A CHI660E electrochemistry workstation was used for all electrochemical measurements (CH Instruments, Shanghai, China). A glass carbon electrode (GCE, 3.0 mm in diameter) functioned as the working electrode in the traditional three-electrode system. A calomel electrode (3 M KCl) served as the reference electrode, and a platinum wire served as the counter electrode. At room temperature, all electrochemical studies were conducted.

Reagents: Tea branches were collected from local tea plantations (Ya’an, China). Norfloxacin (NOR, >98%), enrofloxacin (ENR, >98%) and ciprofloxacin (CIP, >98%) were supplied from Shanghai Aladdin Biochemical Technology Co., Ltd. (Shanghai, China). O-phenylenediamine (o-PD, 98%), o-aminophenol (o-AP, 99%) and chitosan were purchased from Shanghai Aladdin Biochemical Technology Co., Ltd. Food samples (milk, honey and pork) were purchased from the local market (Ya’an, China). The content of the acetate buffer (AB) was 0.1 M (including 0.1 M HAc and 0.1 M NaAc). Throughout this work, ultrapure water with a resistivity of over 18.25 MΩ·cm^−1^ was employed. The other compounds utilized for this experiment were at least analytical grades.

### 2.2. Preparation of Tea Tree Branch Biochar

The tea branch biochar was prepared by high-temperature carbonization treatment and chemical activation method with slight modification [27]. The dried tea branches were washed, crushed and sieved, and then coked for 2 h in an N_2_-atmosphere at 600 °C. The obtained biochar particles were marked as “TBC”.

The as-prepared biochar particles were then chemically activated. For chemical activation, the TBC (1 g) were mixed with different concentrations of K_2_CO_3_ (The quality ratio of samples and K_2_CO_3_ from 1:1 to 1:3) at room temperature, and then dried in an oven at 80 °C. The dried samples are activated in a tubular furnace and the required burning temperatures (650 °C, 700 °C, 750 °C, 800 °C and 850 °C) and burning time (1 h, 1.5 h, 2 h, 2.5 h and 3 h) remain unchanged. After the sample was cooled to room temperature, it was boiled with 0.1 mol/L hydrochloric acid, and then repeatedly washed with hot distilled water until the filtrate became neutral, and then dried at 105 °C. The obtained product was marked as “K-TBC”.

### 2.3. Preparation of the Biochar-Modified GCE

Prior to modification, the GCE was successively polished with 0.3 μm and 0.05 μm Al2O3 slurry on a polishing cloth, followed by a 5-min ultrasonic wash in ultrapure water [28]. The bare electrode was repeatedly cycled between −0.2 and 0.6 V (scan rate, 50 mV/s) prior to the voltammetric test in order to gather reliable responses. To obtain the TBC/GCE, 5 μL of the equally mixed 3 mg TBC and 1 mL of the chitosan-acetic acid solution (the mass concentration of chitosan ranged from 0.1% to 0.5%) was poured onto the naked glassy carbon electrode (the loading of TBC from 1 μL to 6 μL). The solution was then thoroughly dried at room temperature.

### 2.4. Preparation of the MIPs Electrochemical Sensor

Figure 1 shows the preparation procedure of the MIP/TBC/GCE sensor. TBC/GCE was immersed in 10.0 mL AB solution (pH = 5.2) containing 15.0 mM o-PD and 15.0 mM o-AP and 5.0 mM NOR, and 30 segments were scanned by cyclic voltammetry (CV) under the potential range of 0–1.2 V to obtain electrically polymerized membranes [25]. After the electro polymerization, the polymer-modified electrode was incubated in the mixed solution of NaOH (0.1 mol·L^−1^) and 100% ethanol (V:V = 1:3) solution for 12 min to disconnect the hydrogen bond and remove NOR, thus obtaining the MIP/TBC/GCE sensor. As a blank control trial, the non-imprinted electrode (NIP/TBC/GCE) was prepared in the same way as the imprinted electrode except that no template molecule NOR was added.

The effects of electropolymerization solution (from 3.0 to 5.5), the ratio of template molecules to functional monomers (1:15:15, 2:15:15, 3:15:15, 4:15:15, 5:15:15), the molar ratio of oPD to oAP (2:1, 1:1, 1:2, 1:3, 1:4), scanning segments, scanning rate, and elution time on the detection performance of MIP/TBC/GCE were investigated using one-factor-at-a-time experimentation.

### 2.5. Electrochemical Measurement and NOR Detection

Cyclic voltammetry (CV) and differential pulse voltammetry (DPV) were used to characterize the preparation process of the sensor. CV experiments were performed in 1 M KCl solution containing a 5 mM [Fe (CN)_6_]^3−/4−^ form −0.2~0.6 V at the scan rate of 50 mV s^−1^. DPV experiments were performed in 5 mM [Fe (CN)_6_]^3−/4−^ containing 1 M KCl with a pulse amplitude of 0.05 V, pulse width of 0.05 s and pulse period of 0.5 s. For NOR detection (the optimization of partial experimental conditions and properties study) the sensors were incubated with test solutions containing different concentrations of NOR for 4 min, washed carefully with ultrapure water and then immersed in 5 mM [Fe (CN)_6_]^3−/4−^ containing 1 M KCl to record the current responses by differential pulse voltammetry (DPV). The current signal difference (Δ*I*) was calculated according to the following formula:

$$\Delta I=I_{0}-I_{1}$$

where *I*_0_ and *I*_1_ represent the peak current before and after incubation with NOR, respectively.

### 2.6. Sample Preparation

In this study, samples such as milk, honey and pork were pre-processed and tested according to standard procedures to evaluate the suitability of the MIP/TBC/GCE sensor for the detection of NOR in food. Each sample was spiked with three different doses of NOR for spike recovery experiments (6.0, 30.0 and 60.0 nM).

We placed 2 g of spiked milk and pork samples in a 50 mL centrifuge tube, then 5 mL of 10% trichloroacetic acid-acetonitrile solution was added, followed by thorough mixing. After centrifuging at 10,000× *g* rpm for 3 min to remove the protein, the milk sample supernatant was concentrated in a nitrogen stream at 40 °C, redissolved in AB solution (pH = 5.0), and then filtered through a 0.22 μm filter to obtain samples for analysis.

Then, 2 g of spiked honey samples was placed in a 50 mL centrifuge tube, and 20 mL of phosphate buffer solution was added followed by thorough mixing. After centrifuging at 10,000× *g* rpm for 5 min, the supernatant of honey sample is purified in HLB solid phase extraction column, concentrated in nitrogen stream at 40 °C, and redissolved in AB solution (pH = 5.0). Finally, the samples were filtered through a 0.22 μm filter for analysis.

All the experiments were carried out in triplicate.

### 2.7. Statistical Analysis

The data were subjected to analysis of variance, and the means were evaluated using Duncan’s multiple range test (ANOVA, Abacus Concepts, Inc., Piscataway, NJ, USA), with *p* < 0.05 considered significant. The results are reported as the mean ± standard deviation based on multiple samples (*n* = 3).

## 3. Results and Discussion

### 3.1. Characterization of Materials and Polymers

**Figure 2:**
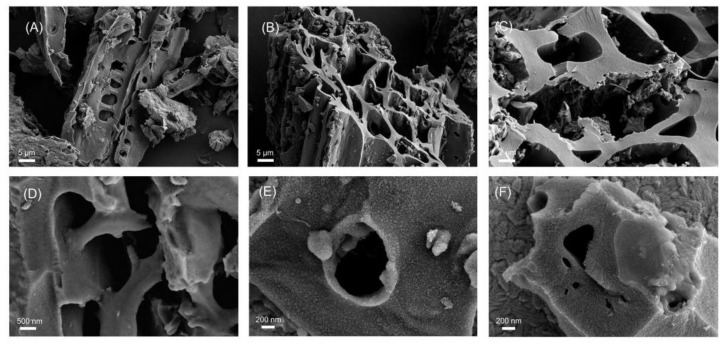
SEM images of TBC (**A**), K-TBC (**B**,**C**), K-TBC/ITO (**D**), NOR-MIP/K-TBC/ITO (**E**) and MIP/K-TBC/ITO (**F**).

Scanning electron microscopy (SEM) was used to describe the morphology of TBC, K-TBC, K-TBC/ITO, NOR-MIP/K-TBC/ITO, and MIP/K-TBC/ITO. In Figure 2A, the TBC sample has a long tubular structure, which can maintain the basic skeleton and organizational structure of plant raw materials without many pores. After the activation of K_2_CO_3_, the K-TBC surface has the pores which were different sizes and different shapes, and the diameter is about 20 nm–10 μm. In addition, its pore structure extends to the interior, with more micropores and a small amount of granular texture (Figure 2B,C). This may be attributed to the removal of hydrogen, oxygen atoms in the pyrolysis process, and the etching of potassium. Other researchers have observed similar findings [29,30,31].

Compared with Figure 2C,D, it can be seen that the surface of K-TBC/ITO is smoother than that of K-TBC, which may be due to the addition of chitosan as a fixative. However, when a molecularly imprinted membrane layer aggregates, the initially smooth surface becomes rough, the hole wall is slightly thickened, and the surface is bright and evenly distributed with white particles (Figure 2E). MIP/K-TBC/ITO was obtained after the modified electrode was immersed in the elution solution to remove the template molecules. Cracks appeared on the surface of the imprinted membrane, and cavities were released (Figure 2F). These results indicate that the preparation process of modified electrode is successful.

**Figure 3:**
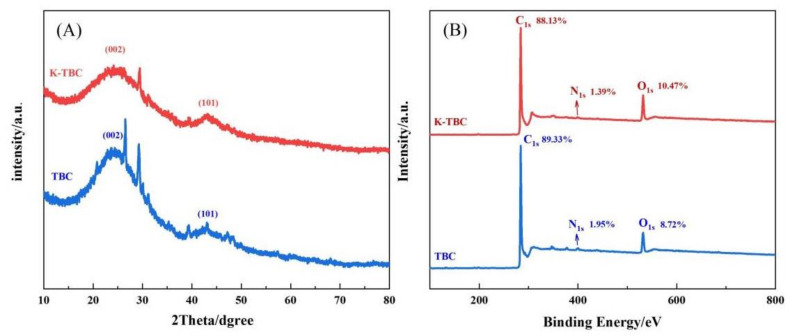
(**A**) XRD patterns and (**B**) The wide XPS survey spectra of K-TBC and TBC.

The X-ray diffraction (XRD) was used to characterize the crystal structure of biochar. Figure 3A shows the X-ray diffraction (XRD) characterization of K-TBC and TBC. Two broad diffusional diffraction peaks on curve a and b are located at around 23.8° and 43.6°, which correspond respectively to the (002) and (101) plane reflection of graphite-carbon structure, it confirms the amorphous carbon structure of K-TBC and TBC accompanied by local graphitization [32,33]. The samples all contain strong miscellaneous peaks, indicating that there are a small number of foreign bodies in the samples, which may be caused by the sample preparation process being insufficiently rigorous. In addition, the intensity of peak (002) can reflect the degree of graphitization [34], indicating that the degree of graphitization of activated K-TBC decreases, indicating that the activated biochar graphite microcrystal disorder increases, the carbon structure tends to be disordered, and the activity of surface atoms is enhanced, which is easy to form a more developed pore structure [35]. In addition, the (101) planes show a higher degree of interlayer packing, which can effectively increase the electrical conductivity of the material. The surface element compositions and chemical valence states of TBC and K-TBC were studied by X-ray photoelectron spectroscopy (XPS). As can be seen from Figure 3B, the total XPS spectra of TBC and K-TBC are similar. K-TBC has three peaks at 285.17 eV, 400.69 eV and 533.02 eV, which are attributed to C1s, O1s and N1s, and their element ratios are 88.13%, 10.47% and 1.39%, respectively. The data showed that the activation of potassium carbonate increased the mass fraction of O on the surface of tea branch biochar, but decreased the mass fraction of C and N at the same time. The reason may be that the potassium carbonate and its decomposition products react with the C in TBC to generate CO_2_, CO and other gases, which escape, consuming a large amount of C element, increasing the relative content of O element and increasing the oxygen-containing groups on the surface [36].

**Figure 4:**
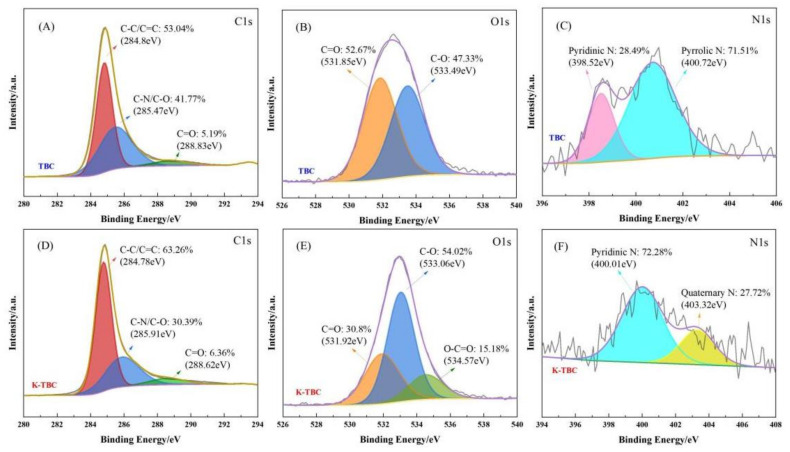
XPS survey spectra of C1s ((**A**) TBC and (**D**) K-TBC), O1s ((**B**) TBC and (**E**) K-TBC) and N1s ((**C**) TBC and (**F**) K-TBC).

In order to further explore the effect of activation on the functional groups of biochar, XPS spectra of TBC and K-TBC were fitted by peak separation. Figure 4A shows that there are three peaks in the C1s spectrum of TBC, which are 284.8, 285.47 and 288.83 eV respectively, and the corresponding functional groups are C-C/C=C, C-N/C-O and C=O [37]. In its O1s spectrum (Figure 4B), C=O (531.85 eV) and C-O (533.49 eV) can be observed [38]. The N1s spectrum of TBC in Figure 4C shows that nitrogen is mainly in the form of pyridine nitrogen (398.52 eV) and pyrrole nitrogen (400.72 eV) [39]. After activation by K_2_CO_3_, C-C/C=C and C=O functional groups increased significantly (Figure 4D), and the presence of a peak of O-C=O (534.57 eV) was observed in the O1s spectrum of K-TBC (Figure 4E) [40]. In addition, the nitrogen functional groups in Figure 4F also changed significantly, with the proportion of pyrrole nitrogen increasing significantly and the presence of graphite nitrogen (403.32 eV) [41]. The high proportion of graphite nitrogen helps to increase the conductivity of the material, and the pyrrole nitrogen and C=O, C-O and -COOH oxygen-containing groups on the surface of the material are conducive to increasing the wettability of porous active biochar in electrolyte solution, thus increasing the contact surface area of ions and enhancing the interaction between porous biochar and electrolyte [42].

### 3.2. Characterization of Electrochemical Properties

**Figure 5:**
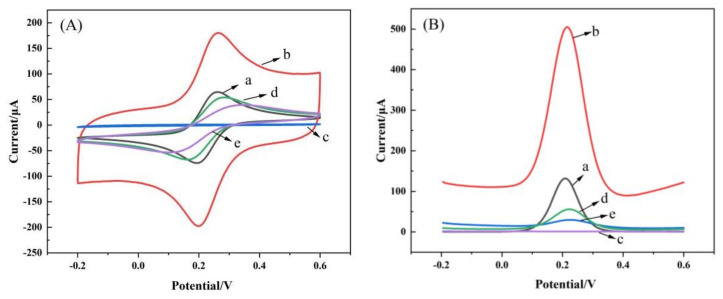
CV (**A**) and DPV (**B**) plots of of 5 mM [Fe (CN)_6_]^3−/4−^ in 1 M KCl solution at GCE (a), TGC/GCE (b), NOR−MIP/TGC/GCE (c), MIP/TGC/GCE (d) and NORad−MIP/TGC/GCE (e).

K_3_[Fe (CN)_6_] is the most commonly used analytical reagent in electrochemical methods. Due to its excellent oxidized and reduced properties, K_3_[Fe (CN)_6_] can be used to indirectly characterize the changes of conductance properties during the preparation of sensors by cyclic voltammetry (CV) and differential pulse voltammetry (DPV). As shown in Figure 5A, the electric and oxidized and reduced current and current area of the modified K-TBC (curve b) are larger than those of the bare electrode (curve a), because BC has the advantages of multiple active sites and large specific surface area in the electrochemical oxidation process, and thus has strong conductive properties. The CV current response after polymerized MIP (curve d) is lower than that before polymerization (curve c), because MIP is a poorly conductive polymer, which hinders the electron conduction of [Fe (CN)_6_]^3−/4−^ [43]. The NOR in MIPs eluted with organic solvents formed molecule-imprinted pores that facilitated electron conduction, so the current response of the eluted MIPs (curve e) was stronger than that of the pre-eluted MIPS (curve d). Curves e and c show that the current response of the eluted MIPs is still lower than that of K-TBC/GCE of the unpolymerized MIPs, which indicates that there is still polymer on the surface of the eluted electrode. This is illustrated by the DPV in Figure 5B.

### 3.3. Optimization of Experimental Conditions

#### 3.3.1. Optimization of Conditions for the Fabrication of the TBC/GCE

**Figure 6:**
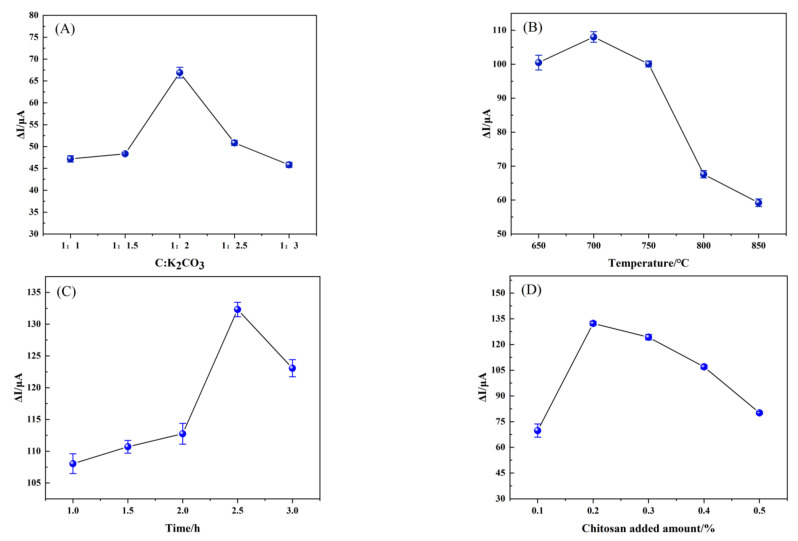

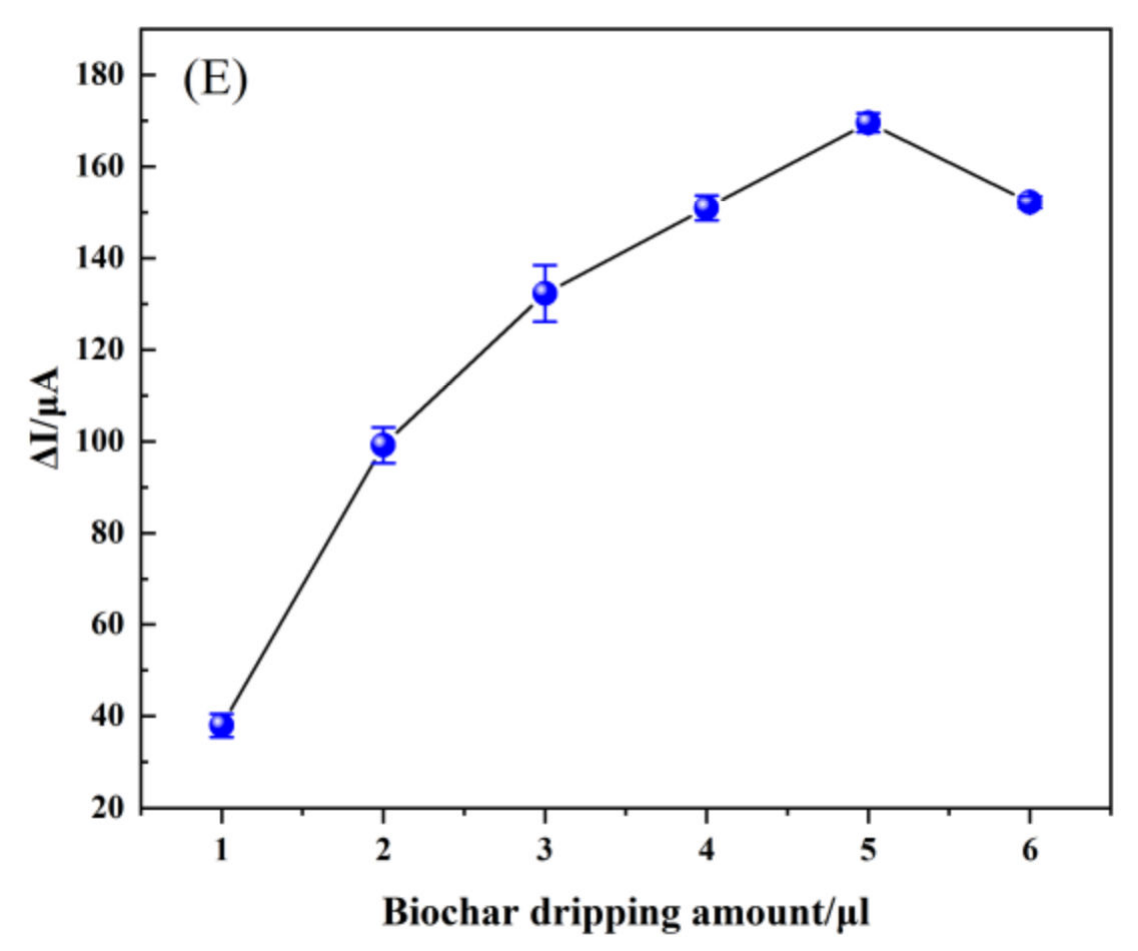
The plots for the relationship of (**A**) the ratio of biochar and activator, (**B**) the firing temperature of TBC, (**C**) the roasting time of TBC, (**D**) chitosan addition amount and (**E**) TBC dripping amount on the current difference (ΔI) by CV in 5 mmol·L^−1^ [Fe (CN)_6_]^3–/4−^ (1.0 mol·L^−1^ KCl).

The structure and properties of biomass carbon materials vary with the products obtained by different preparation processes and different conditions, including pretreatment methods, carbonization temperature, heating rate, cracking time and gas atmosphere [20,44]. The ratio of biochar to activator, the calcination temperature, the calcination time, the amount of chitosan added, and the loading of TBC were optimized in order to increase the electrical conductivity of TBC/GCE, with the current difference between the electrode before and after modification being measured by CV (−0.2 V–0.6 V, 50 mV·s^−1^) in probe solution as the index. According to the experimental results, when the ratio of biochar to activator is 1:2 (Figure 6A), the roasting temperature of TBC is 700 °C (Figure 6B), the roasting time of TBC is 2.5 h (Figure 6C), the addition of chitosan is 0.2% (Figure 6D), and the loading of TBC is 5 μL (Figure 6E), the prepared TBC/GCE has the highest current response. In addition, with the increase of the concentration of TBC suspension, the response current value also increases, but the increase of the concentration is not conducive to the subsequent binding with the molecular-imprinted copolymer. Therefore, 3 mg/mL TBC suspension concentration was selected to prepare an MIP/TBC/GCE sensor.

#### 3.3.2. Optimization of Conditions for the Fabrication of the MIP/TBC/GCE

It is important to optimize sensor preparation conditions, including background pH of electropolymerization, ratio of template molecule to functional monomer, ratio of composite functional monomer, scanning segment, scanning rate and elution time. The modified electrodes under various circumstances were incubated in 0.1 μM NOR solution for DPV detection in the probe solution.

**Figure 7:**
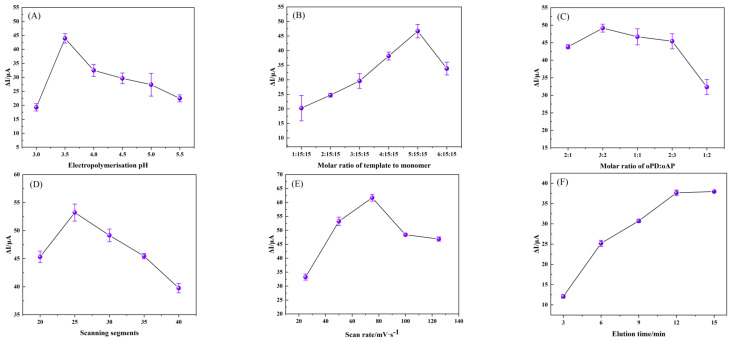
The plots for the relationship of (**A**) background pH of electropolymerization, (**B**) molar ratio of template to monomer, (**C**) molar ratio of oPD:oAP, (**D**) scanning segments, (**E**) scan rate and (**F**) elution time on the current difference (ΔI) by DPV in 5 mmol·L^−1^ [Fe (CN)_6_]^3−/4−^ (1.0 mol·L^−1^ KCl). ΔI = I_0_ − I_1_, I_0_ and I_1_ were the peak currents obtained from MIP/TBC/GCE before and after being incubated with 0.1 μM NOR.

The interaction between the template molecule and the functional monomer, as well as the structural stability of the membrane bearing the molecular imprint, are both impacted by the pH level of the polymerization solution. Therefore, choosing a suitable pH level electropolymerization solution has a certain influence on the performance of the sensor. Polymerization solutions with pH levels of 3.0, 3.5, 4.0, 4.5 and 5.0 were created to study the effect of pH values on the performance of composite molecularly imprinted membranes. The experimental results show that the current response difference is the largest when pH = 3.5 (Figure 7A), indicating that this pH is most favorable for the interaction of oPD, oAP and NOR, which is conducive to the formation of more binding sites [26].

An important condition for the preparation of molecularly imprinted electrochemical sensors by electropolymerization is to determine the amount of template molecules and functional monomers. Keeping other conditions unchanged, with a fixed total functional monomer concentration of 30 mM (oPD:oAP = 1:1), the developed sensor response signal was tested at different ratios of NOR and total functional monomer (1:15:15, 2:15:15, 3:15:15, 4:15:15, 5:15:15, 6:15:15, Figure 7B), when the ratio is 5:15:15, the largest current difference, but when the ratio was lower or higher than 5:15:15, the current response decreased significantly, probably because during the polymerization process, if the concentration of functional monomers was low, the bound NOR was less, and the number of imprinted sites decreased. With a high concentration of functional monomers, the copolymer film becomes denser, impeding template molecule removal and adsorption as well as electron transfer on the electrode surface [45]. In addition, the performance of MIP is also affected by the monomer molar ratio. In order to obtain the optimal copolymer for forming imprinted sites, keeping the rest of the conditions the same, a fixed total functional monomer concentration of 30 mM with different molar ratios of monomers (oPD:oAP, 2:1, 3:2, 1:1, 2:3, 1:2) is maintained. Figure 7C is the current difference before and after the electrode adsorption, and it can be seen that the maximum current response is obtained under the monomer with the ratio of 3:2.

Both the scan segment and electropolymerization scan rate are significant factors in the manufacturing of MIP/TGC/GCE, affecting the thickness and density of the molecularly imprinted polymer, respectively. It was found in the experiment that the 25 scan segments showed relatively high sensitivity (Figure 7D), which may be due to the small number of polymerizations, which resulted in fewer imprinted sites for NOR binding, and higher polymerization times would lead to more polymer. A thick film leaves the imprinted site incompletely exposed and affects NOR recombination. The effect of scan rate on electropolymerization is illustrated in Figure 7E. For one thing, at slightly slower scan rates, dense MIP layers are formed, making the removal of NOR to form imprinted sites less feasible. For another thing, the non-compact and rough MIP layers formed at higher scan rates may be affected by the eluent.

The removal of all template molecules is a critical step in the fabrication of molecularly imprinted electrochemical sensors. The elution time was investigated by immersing the MIP sensor in NaOH (0.1 mol·L^−1^) and 100% ethanol (V:V = 1:3) solution for different times. Figure 7F depicts the elution profile of MIP/TGC/GCE. It is not difficult to see that as the elution time increases, the current increases evenly until it stabilized after more than 12 min. This indicates that the template molecule has been entirely removed from the MIP. Thus, 12 min is the optimal time for template removal.

#### 3.3.3. Optimization of Conditions for the Fabrication of the MIP/TBC/GCE

**Figure 8:**
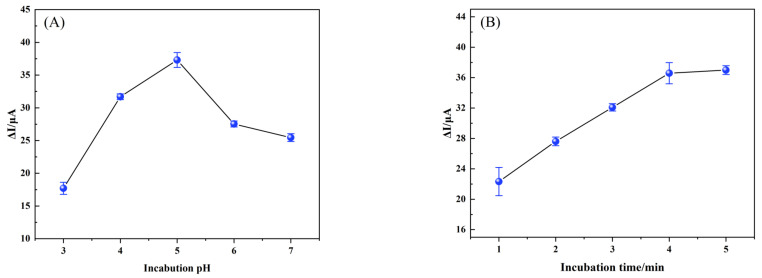
The plots for the relationship of (**A**) Incubation pH (3.0, 4.0, 5.0, 6.0, 7.0) and (**B**) Incubation time (1, 2, 3, 4, 5 min) on the current difference (ΔI) by DPV in 5 mmol·L^−1^ [Fe (CN)_6_]^3−/4−^ (1.0 mol·L^−1^ KCl). ΔI = I_0_ − I_1_, I_0_ and I_1_ were the peak currents obtained from MIP/TBC/GCE before and after incubated with 0.1 μM NOR.

The same concentration of NOR solution was used to study the effect of incubation time and pH on the electrochemical performance of the sensor. As shown in Figure 8A, as the pH increased from 3.0 to 7.0, the peak current difference first increased and then decreased, and the maximum peak current difference (ΔI) was obtained at pH 5.0, which was considered to be the optimum pH for the experiment.

The modified electrodes were incubated in 0.1 μM NOR solution for different times, and DPV detection was performed in K_3_[Fe (CN)_6_] solution. Figure 8B shows that as absorption time increases, so does the peak current difference, and the current is almost stable after 4 min. This trend indicates that the adsorption capacity has reached its limit. As a result, an incubation time of 4 min is ideal.

### 3.4. Study on the Properties of the MIP/TBC/GCE Sensor

#### 3.4.1. Linear Range and Limit of Detection

**Figure 9:**
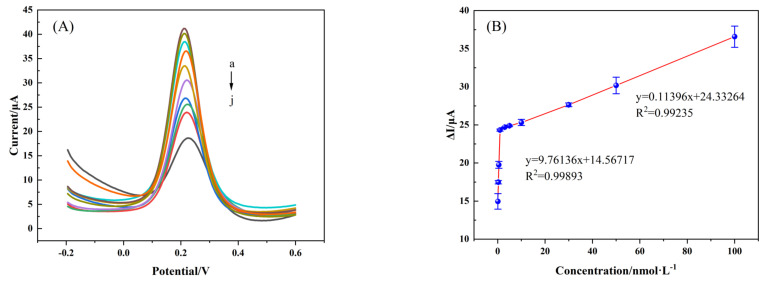
(**A**) DPV plots in 5 mmol·L^−1^ [Fe (CN)_6_]^3−/4−^ (1.0 mol·L^−1^ KCl) obtained from MIP/TBC/GCE after incubation with different concentrations of NOR (0.1, 0.3, 0.5, 1, 3, 5, 10, 30, 50, 100 nM) (a → j); (**B**) The relationship between current difference (ΔI) and the concentrations of NOR. ΔI = I_0_ − I_1_, I_0_ and I_1_ were the peak currents obtained from electrodes before and after incubation with various concentrations of NOR solution.

To monitor the effectiveness of the constructed sensor, the MIP/TBC/GCE sensor was tested for different concentrations of NOR solutions (0.1, 0.3, 0.5, 1, 3, 5, 10, 30, 50, 100 nM) under optimal experimental conditions. DPV experiments were performed and Figure 9A shows that the peak current decreases with increasing NOR concentration. While Figure 9B further indicates that a two-stage linear association between ΔI and NOR concentration. In the low concentration NOR solution range (0.1–0.5 nM), the regression equation is ΔI (μA) = 9.76136 c (nM) + 14.56717 (R^2^ = 0.99893), and at high concentration (0.5–100 nM), the regression equation is ΔI (μA) = 0.11396 c (nM) + 24.332642 (R^2^ = 0.99235), ΔI is the difference in current between before and after NOR adsorption by the MIP/TBC/GCE sensor, and c represents the concentration of NOR standard solution. This indicates that the local concentration on the sensor surface is rapidly depleted at low NOR solution concentration, resulting in a high sensor response sensitivity, while saturation occurs at high concentration conditions, and the kinetic adsorption rate is significantly reduced, resulting in a decrease in the slope to 0.11396 [46]. When calculated according to formula 3S_b_/K, the limit of detection (LOD) is about 0.028nM, where S_b_ is the standard deviation of the blank value’s six measured values (ΔI) (NOR concentration close to the blank value but still current response), and K is the calibration curve’s slope for low concentrations.

#### 3.4.2. Selectivity, Reproducibility and Stability

**Figure 10:**
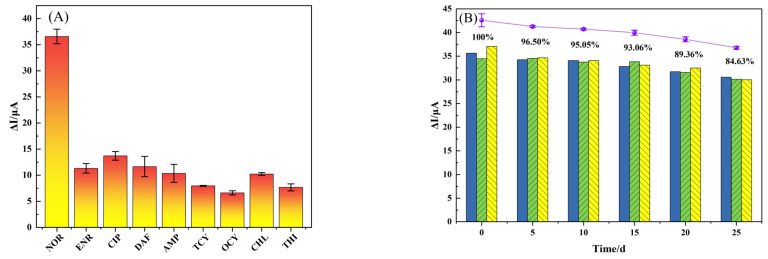
(**A**) the selectivity and (**B**) stability of MIP/TBC/GCE sensor.

Shape, size and functional group are the most critical factors for the recognition of template molecules by the imprinted cavity [47]. Therefore, it is useful to examine enrofloxacin (ENR), ciprofloxacin (CIP), and danofloxacin (DAF), which are similar in structure to NOR, then ampicillin (AMP), tetracycline (TCY), oxytetracycline (OCY), chloramphenicol (CHL) and thiamphenicol (THI) —which are quite different from NOR in structure— to study selectivity. As shown in Figure 10A, among different antibiotics at the same concentration, the sensor exhibited the highest current response to NOR, and a slight current response to structural analogs, which were slightly higher than those of non-structural analogs. It shows that the sensor has good recognition specificity and selectivity for NOR.

Interference experiments were conducted to study the effects of common components such as metal ions, carbohydrates and amino acids in food matrices on the detection of NOR by MIP/TBC/GCE sensors. When the change of ΔI is less than 5%, it can be considered that there is no significant impact on the test object [48,49]. The results are shown in Table 1. When the concentration of NOR is 0.1 μM, high concentrations of Na^+^, K^+^, Ca^2+^, Fe^2+^, Al^3+^, NO_3_^−^, Cl^−^, as well as glucose, phenylalanine, cysteine and lysine, etc, are seen. The ΔI of NOR was not significantly affected, and the relative errors were all less than ±4.1%.

The reproducibility and stability of the MIP/TBC/GCE sensor were investigated by the change in ΔI in 0.1 μM NOR standard solution. The relative standard deviation (RSD) for different sensors (*n* = 5) and the same sensor (*n* = 5) were 0.92% and 1.36%, respectively (Table 2). According to the above results, the MIP/TBC/GCE sensor exhibited satisfactory reproducibility.

To study the stability of the constructed sensor, the MIP/TBC/GCE sensor was stored for 0, 5, 10, 15, 20, and 25 days for the detection of the same concentration of NOR, and the ΔI was recorded by DPV. After preparation, the sensor was placed in a nitrogen-filled airtight bag and stored in a 4 °C refrigerator. As the storage days increased, the peak current of the modified electrode for detecting NOR decreased, and on the 25th day, the peak current was 84.63% of the initial current (Figure 10B), indicating that the developed MIP/TBC/GCE sensor has excellent stability. The decrease in current response may be due to the damage of the recognition site due to oxidation of the film, or the film peeling off the electrode surface due to poor adhesion of the film [50].

#### 3.4.3. Application of Real Sample Detection

Three animal-derived foods, milk, honey and pork, which are prone to NOR residues in actual production, were selected as samples for NOR detection to evaluate the applicability of the sensor in practical applications. It can be seen from Table 3 that no NOR residues were detected in the commercially available milk, honey and pork samples, indicating that the samples were not contaminated by NOR. At the same time, the standard addition method was used for quantitative analysis, and the recoveries of milk samples were 95.2–102.0%, the recoveries of honey samples were 96.6–102.0%, and the recoveries of pork samples were 85.9–101.7%. All results showed that the recovery and precision of this sensor were ideal and it is feasible to detect NOR in milk, honey and pork samples.

Comparing the MIP/TBC/GCE sensor prepared in this study with published NOR detection methods in Table 4, it can be noted that the proposed sensor exhibits higher sensitivity than most other sensors and its incubation time was at a medium level.

## 4. Conclusions

In summary, a simple and low-cost biochar molecular-imprinted electrochemical sensor fabricated from discarded tea branch precursors was constructed for the detection of NOR. Tea branch biochar provides porous structure and large specific surface area to enhance the conductivity of the sensor. A molecular-imprinted layer provides specific recognition ability to further improve the sensitivity of the sensor, which makes the lower limit of detection (LOD) 0.028 nM (S/N = 3) and the linear range wider (0.1–100 nM). At the same time, it also shows excellent performance, including short detection time, good selectivity and stability. In addition, the sensor was able to detect NOR in milk, honey and pork, with recovery rates between 85.90% and 101.71%, and RSD less than 5%, which proved its potential in the detection of NOR residues in animal-derived foods. Efforts should be made to commercialize the sensor and provide a new idea for food quality monitoring.

## Figures and Tables

**Table 1:** Effects of Different Interfering Substances on NOR Detection by MIP/TBC/GCE Sensors.

Interfering Substances	Concentration Multiple	Relative Error (%)	Interfering Substances	Concentration Multiple	Relative Error (%)
Na^+^	500	−1.21	Fe^2+^	200	−3.21
Cl^−^	500	−1.21	glucose	80	−3.72
K^+^	500	+3.26	Phenylalanine (Phe)	50	−0.96
NO_3_^−^	500	+3.26	Cysteine (Cys)	50	−4.08
Ca^2+^	200	−2.00	Lysine (Lys)	50	−2.76

**Table 2:** The reproducibility of MIP/TBC/GCE sensor.

	ΔI_1_/(μA)	ΔI_2_/(μA)	ΔI_3_/(μA)	ΔI_4_/(μA)	ΔI_5_/(μA)	RSD (%)
Different electrodes	34.9	35.43	35.31	35.36	35.92	0.92
Same electrode	35.48	34.97	34.4	34.31	34.27	1.36

**Table 3:** Results of NOR detection in real food samples (*n* = 3).

Samples	Added (nM)	ΔI_0_ (μA)	ΔI_1_ (μA)	Found (nM)	Recovery (%)	RSD (%)
Milk-1	0	4.84	-	nd ^a^	-	-
Milk-2	6	-	^29.87^	^6.12^	102.0 ± 1.21	4.05
Milk-3	30	-	^32.65^	^30.51^	101.7 ± 0.10	0.31
Milk-4	60	-	^35.68^	^57.10^	95.2 ± 0.39	1.08
Honey-1	0	7.80	-	nd ^a^		
Honey-2	6	-	32.83	^6.12^	102.0 ± 0.07	0.22
Honey-3	30	-	35.56	30.08	100.3 ± 0.14	0.40
Honey-4	60	-	38.74	57.97	96.6 ± 0.05	0.12
Pork-1	0	8.80	-	nd ^a^	-	-
Pork-2	6	-	33.72	5.15	85.9 ± 0.04	0.11
Pork-3	30	-	36.61	30.51	101.7 ± 0.3	0.83
Pork-4	60	-	39.80	58.5	97.5 ± 0.60	1.50

nd ^a^ means not detected.

**Table 4:** Linear range and LOD of MIP/TBC/GCE compared with other previously reported sensors.

Modified Electrodes	Methods	Samples	Linear Range (mol L^−1^)	LOD (mol L^−1^)	Incubation Time (min)	Ref.
MWCNT-CPE/pRGO-ANSA/Au	DPV	drug tablet	3 × 10^−8^–5 × 10^−5^	1.6 × 10^−8^	3.5	[47]
fMWCNTs-MIP/GCE	DPV	drug tablet and rat plasma	3 × 10^−9^–3.13 × 10^−6^	1.58 × 10^−9^	9.0	[48]
MIP/CoFe-MOFs/AuNPs/GCE	DPV	drug capsules and milk	5 × 10^−12^–6 × 10^−9^	1.31 × 10^−13^	3	[49]
MIP/Cu-N-C/GCE	DPV	drug capsules	1 × 10^−8^–5 × 10^−7^, 5 × 10^−7^–1 × 10^−4^	3.33 × 10^−9^	1.5	[50]
Sensor in this work	DPV	milk, honey and pork	1 × 10^−10^–5 × 10^−10^, 5 × 10^−10^–1 × 10^−7^	2.8 × 10^−11^	4	This work

## Data Availability

Data are contained within the article.

## References

[1] Norrby S.R., Jonsson M. (1983). Antibacterial activity of norfloxacin. Antimicrob. Agents Chemother..

[2] Lucas C., Hérida R.N.S. (2015). Review of Properties and Analytical Methods for the Determination of Norfloxacin. Crit. Rev. Anal. Chem..

[3] Califf R.M. (2019). Oral Fluoroquinolones. J. Am. Coll. Cardiol..

[4] Ribeiro N.V., Melo R.G., Guerra N.C., Guerra N.C., Nobre A., Fernandes R.M., Pedro L.M., Costa J., Costa F.J., Caldeira D. (2021). Fluoroquinolones are associated with increased risk of aortic aneurysm or dissection: Systematic review and meta-analysis. Semin. Thorac. Cardiovasc. Surg..

[5] Huang L.L., Mo Y.M., Wu Z.Q., Rad S., Song X.H., Zeng H.H., Bashir S., Kang B., Chen Z.B. (2020). Occurrence, distribution, and health risk assessmentof quinolone antibiotics in watcr, sediment, and fish species of Qingshitan reservoir, South China. Sci. Rep..

[6] The Ministry of Agriculture of the People’s Republic of China (2015). Bulletin of the Ministry of Agriculture of the People’s Republic of China.

[7] The Ministry of Agriculture of the People’s Republic of China (2021). Circular on the Monitoring Results of Veterinary Drug Residues in Animals and Animal Products in 2020.

[8] Weng R., Sun L.S., Jiang L.P., Li N., Ruan G.H., Li J.P., Du F.Y. (2019). Electrospun Graphene Oxide–Doped Nanofiber-Based Solid Phase Extraction Followed by High-Performance Liquid Chromatography for the Determination of Tetracycline Antibiotic Residues in Food Samples. Food Anal. Methods.

[9] Zou W., Wen X.K., Xia C.H., Nie L., Zhou Q., Chen X.C., Fang C.Y., Wang Y.C., Zhang L. (2018). LC-Q-TOF-MS based plasma metabolomic profile of subclinical pelvic inflammatory disease: A pilot study. Clin. Chim. Acta.

[10] Kanda M., Kusano T., Kanai S., Hayashi H., Matushima Y., Nakajima T., Takeba K., Sasamoto T., Nagayma T. (2010). Rapid Determination of Fluoroquinolone Residues in Honey by a Microbiological Screening Method and Liquid Chromatography. J. AOAC Int..

[11] Moonsun J., Insook R.P. (2008). Quantitative detection of tetracycline residues in honey by a simple sensitive immunoassay. Anal. Chim. Acta.

[12] Patrícia B.D., Romeu C.R., Orlando F. (2018). A new and simple method for the simultaneous determination of amoxicillin and nimesulide using carbon black within a dihexadecylphosphate film as electrochemical sensor. Talanta.

[13] Manel R., Mabrouk B.B., Youssef S. (2017). Electrochemical determination of levofloxacin antibiotic in biological samples using boron doped diamond electrode. J. Electroanal. Chem..

[14] Ahmed W., Xu T.W., Mahmood M., Nunez-Delgado A., Ali S., Shakoor A., Qaswar M., Zhao H.W., Liu W.J., Li W.D. (2022). Nano-hydroxyapatite modified biochar: Insights into the dynamic adsorption and performance of lead (II) removal from aqueous solution. Environ. Res..

[15] Zhang L., Xu L., Zhang Y.G., Zhou X., Zhang L.T., Yasin A., Wang L.L., Zhi K.K. (2018). Facile synthesis of bio-based nitrogen- and oxygen-doped porous carbon derived from cotton for supercapacitors. RSC Adv..

[16] Kumar A., Upadhyay S.N., Mishra P.K., Mondal M.K. (2022). Multivariable modeling, optimization and experimental study of Cr (VI) removal from aqueous solution using peanut shell biochar. Environ. Res..

[17] Zhou J.Z., Luo A.R., Zhao Y.C. (2018). Preparation and characterisation of activated carbon from waste tea by physical activation using steam. J. Air Waste Manag..

[18] Asadi-Sangachini Z., Galangash M.M., Younesi H., Nowrouzi M. (2019). The feasibility of cost-effective manufacturing activated carbon derived from walnut shells for large-scale CO_2_ capture. Environ. Sci. Pollut. R..

[19] Liang Q.L., Liu Y.C., Chen M.Y., Ma L.L., Yang B., Li L.L., Liu Q. (2020). Optimized preparation of activated carbon from coconut shell and civil for municipal sludge. Mater. Chem. Phys..

[20] Han C., Wang M., Ren Y., Zhang L., Ji Y., Zhu W., Song Y., He J. (2021). Characterization of pruned tea branch biochar and the mechanisms underlying its adsorption for cadmium in aqueous solution. RSC Adv..

[21] Akgül G., Iglesias D., Ocon P., Jiménez E.M. (2019). Valorization of Tea-Waste Biochar for Energy Storage. Bioenerg. Res..

[22] Gui R., Hui J., Guo H., Wang Z. (2018). Recent advances and future prospects in molecularly imprinted polymers-based electrochemical biosensors. Biosens. Bioelectron..

[23] Singh M., Singh S., Singh S.P., Patel S.S. (2020). Recent advancement of carbon nanomaterials engrained molecular imprinted polymer for environmental matrix. Trends Environ. Anal. Chem..

[24] Kong Y., Li X.Y., Ni J.H., Yao C., Chen Z.D. (2012). Enantioselective recognition of glutamic acid enantiomers based on poly (aniline-co-m-aminophenol) electrode column. Electrochem. Commun..

[25] Kong Y., Shan X., Ma J., Chen M., Chen Z. (2014). A novel voltammetric sensor for ascorbic acid based on molecularly imprinted poly(o-phenylenediamine-co-o-aminophenol). Anal. Chim. Acta.

[26] Khumngern S., Thavarungkul P., Kanatharana P., Bejrananda T., Numnuam A. (2022). Molecularly imprinted electrochemical sensor based on poly(o-phenylenediamine-co-o-aminophenol) incorporated with poly(styrenesulfonate) doped poly(3,4-ethylenedioxythiophene) ferrocene composite modified screen-printed carbon electrode for highly sensitive and selective detection of prostate cancer biomarker. Microchem. J..

[27] Pang P., Yan F., Chen M., Li H.Y., Zhang Y.L., Wang H.B., Wu Z., Yang W.R. (2016). Promising biomass-derived activated carbon and gold nanoparticle nanocomposites as a novel electrode material for electrochemical detection of rutin. RSC Adv..

[28] Ye Y., Jian J., Pi F., Yang H.C., Liu J., Zhang Y.Z. (2018). A novel electrochemical biosensor for antioxidant evaluation of phloretin based on cell-alginate/-cysteine/gold nanoparticle-modified glassy carbon electrode. Biosens. Bioelectron..

[29] Zhu L., Zhao N., Tong L.H., Lv Y.Z. (2018). Structural and adsorption characteristics of potassium carbonate activated biochar. RSC Adv..

[30] Madhu R., Veeramani V., Chen S.M. (2014). Heteroatom-enriched and renewable banana-stem-derived porous carbon for the electrochemical determination of nitrite in various water samples. Sci. Rep..

[31] Goswami R., Shim J., Deka S., Kumari D., Kataki R., Kumar M. (2016). Characterization of cadmium removal from aqueous solution by biochar produced from Ipomoea fistulosa at different pyrolytic temperatures. Ecol. Eng..

[32] Yang F., Sun L.L., Xie W.L., Jiang Q., Gao Y., Zhang W., Zhang Y. (2017). Nitrogen-functionalization biochars derived from wheat straws via molten salt synthesis: An efficient adsorbent for atrazine removal. Sci. Total Environ..

[33] Chen L.F., Zhang X.D., Liang H.W., Kong M.G., Guan Q.F., Chen P., Wu Z.Y., Yu S.H. (2012). Synthesis of Nitrogen-Doped Porous Carbon Nanofibers as an Efficient Electrode Material for Supercapacitors. ACS Nano.

[34] Li X.X., Li H.Y., Liu T.T., Hei Y.S., Hassan M., Zhang S.Y., Lin J.X., Wang T.S., Bo X.J., Wang H.L. (2018). The biomass of ground cherry husks derived carbon nanoplates for electrochemical sensing. Sens. Actuators B Chem..

[35] Kong J.J., Zheng Y.J., Xiao L.J., Dai B.L., Meng Y.J., Ma Z.Y., Wang J.H., Huang X.C. (2020). Synthesis and comparison studies of activated carbons based folium cycas for ciprofloxacin adsorption. Colloids Surf. A.

[36] Zhao C., Li Y., He Z., Jiang Y., Li L., Jiang F. (2019). KHCO_3_ activated carbon microsphere as excellent electrocatalyst for VO~(2+)/VO2~+ redox couple for vanadium redox flow battery. J. Energy Chem..

[37] Chen C., Qian S., Yao T.H., Guo J.H., Wang H.K. (2021). Plate-like carbon-supported Fe3C nanoparticles with superior electrochemical performance. Rare Metals.

[38] Wang L.L., Wang J., Ng D., Li S., Zou B.B., Cui Y.X., Liu X.H., El-Khodary S.A., Qiu J.X., Lian J.B. (2021). Operando mechanistic and dynamic studies of N/P co-doped hard carbon nanofibers for efficient sodium storage. Chem. Commun..

[39] Xu J., Liu J.W., Ling P., Zhang X., Xu K., He L.M., Wang Y., Su S., Hu S., Xiang J. (2020). Raman spectroscopy of biochar from the pyrolysis of three typical Chinese biomasses: A novel method for rapidly evaluating the biochar property. Energy.

[40] Anthonysamy S.I., Lahijani P., Mohammadi M., Mohamed A.R. (2022). Alkali-modified biochar as a sustainable adsorbent for the low-temperature uptake of nitric oxide. Int. J. Environ. Sci. Technol..

[41] Tang C.G., Liu Y.J., Yang D.G., Yang M., Li H.M. (2017). Oxygen and nitrogen co-doped porous carbons with finely-layered schistose structure for high-rate-performance supercapacitors. Carbon.

[42] Li X.P., Zhang J.G., Liu B. (2021). A critical review on the application and recent developments of post-modifiedbiochar in supercapacitors. J. Clean. Prod..

[43] Rao H.B., Chen M., Ge H.W., Lu Z.W., Liu X., Zou P., Wang X.X., He H., Zeng X.Y., Wang Y.Y. (2017). A novel electrochemical sensor based on Au@PANI composites film modified glassy carbon electrode binding molecular imprinting technique for the determination of melamine. Biosens. Bioelectron..

[44] Celiktas M.S., Alptekin F.M. (2019). Conversion of model biomass to carbon-based material with high conductivity by using carbonization. Energy.

[45] An J., Li L., Ding Y. (2019). A novel molecularly imprinted electrochemical sensor based on Prussian blue analogue generated by iron metal organic frameworks for highly sensitive detection of melamine. Electrochim. Acta.

[46] Dong X.X., Li M.Y., Feng N.N. (2015). A nanoporous MgO based nonenzymatic electrochemical sensor for rapid screening of hydrogen peroxide in milk. RSC Adv..

[47] Liu Z., Jin M., Cao J. (2017). High-sensitive Electrochemical Sensor for Determination of Norfloxacin and Its Metabolism Using MWCNT-CPE/pRGO-ANSA/Au. Sens. Actuators B Chem..

[48] Liu Z., Jin M., Lu H. (2019). Molecularly imprinted polymer decorated 3D-framework of functionalized multi-walled carbon nanotubes for ultrasensitive electrochemical sensing of Norfloxacin in pharmaceutical formulations and rat plasma. Sens. Actuators B Chem..

[49] Ye C., Chen X., Zhang D. (2021). Study on the properties and reaction mechanism of polypyrrole@norfloxacin molecularly imprinted electrochemical sensor based on three-dimensional CoFe-MOFs/AuNPs. Electrochim. Acta.

[50] Wang Q.T., Cheng S.N., Ren S.F. (2022). Construction of Molecularly Imprinted Voltammetric Sensor Based on Cu-N-C Polyhedron Porous Carbon from Cu Doping ZIF-8 for the Selective Determination of Norfloxacin. Microchem. J..

